# Dual-Energy Computed Tomography of the Lung in COVID-19 Patients: Mismatch of Perfusion Defects and Pulmonary Opacities

**DOI:** 10.3390/diagnostics10110870

**Published:** 2020-10-26

**Authors:** Saif Afat, Ahmed E. Othman, Konstantin Nikolaou, Sebastian Gassenmaier

**Affiliations:** Department of Diagnostic and Interventional Radiology, Eberhard-Karls-University Tuebingen, 72076 Tuebingen, Germany; saif.afat@med.uni-tuebingen.de (S.A.); konstantin.nikolaou@med.uni-tuebingen.de (K.N.); sebastian.gassenmaier@med.uni-tuebingen.de (S.G.)

**Keywords:** dual energy, computed tomography, COVID-19, coronavirus, perfusion, opacity, consolidation, lung

## Abstract

To evaluate contrast-enhanced dual-energy computed tomography (DECT) chest examinations regarding pulmonary perfusion patterns and pulmonary opacities in patients with confirmed COVID-19 disease. Fourteen patients with 24 DECT examinations performed between April and May 2020 were included in this retrospective study. DECT studies were assessed independently by two radiologists regarding pulmonary perfusion defects, using a Likert scale ranging from 1 to 4. Furthermore, in all imaging studies the extent of pulmonary opacities was quantified using the same rating system as for perfusion defects. The main pulmonary findings were ground glass opacities (GGO) in all 24 examinations and pulmonary consolidations in 22 examinations. The total lung scores after the addition of the scores of the single lobes showed significantly higher values of opacities compared to perfusion defects, with a median of 12 (9–18) for perfusion defects and a median of 17 (15–19) for pulmonary opacities (*p* = 0.002). Furthermore, mosaic perfusion patterns were found in 19 examinations in areas with and without GGO. Further studies will be necessary to investigate the pathophysiological background of GGO with maintained perfusion compared to GGO with reduced perfusion, especially regarding long-term lung damage and prognosis.

## 1. Introduction

In December 2019, a novel coronavirus called severe acute respiratory syndrome coronavirus 2 (SARS-CoV-2) emerged in mainland China, leading to pneumonia of different severity [1,2]. This kind of pneumonia was later named “Coronavirus Disease 2019” (COVID-19) by the World Health Organization (WHO). Meanwhile, the outbreak of COVID-19 has developed into a pandemic, affecting almost every country worldwide and leading to millions of infections and hundreds of thousands of deaths. Therefore, in January 2020 the WHO declared a global health emergency [3]. To confine the further spread of this disease, an early diagnosis of COVID-19 is vital. One of the most common and useful diagnostic tools is reverse transcriptase polymerase chain reaction (RT-PCR) [4]. During the course of the pandemic, computed tomography (CT) imaging of the chest has gained increasing importance for diagnosis, the assessment of the extent of pulmonary involvement, the prediction and confirmation of possible complications, and for follow-up [5,6]. Typical CT imaging patterns have been described previously, mainly bilateral ground glass opacities (GGO), interlobular septal thickening, crazy paving, and consolidation [1,6,7,8,9,10,11,12,13,14,15].

Despite parenchymal lung damage, thromboembolic incidents, pulmonary and non-pulmonary, have been described [16,17,18,19,20,21,22]. So far, the exact pathogenesis of thromboembolic incidents and perfusion disorders due to COVID-19 is unclear.

For a more detailed CT diagnosis, the implementation of dual-energy CT (DECT) allows the possibility of the creation of iodine maps. These maps can be used to quantify perfusion disorders within lung parenchyma—e.g., in pulmonary embolism or pulmonary hypertension [23,24,25,26,27,28]. In a previous study by Lang et al., possible pulmonary vascular manifestations of COVID-19 using DECT were demonstrated, including mosaic perfusion, regional hyperemia, as well as oligemia [29,30]. However, the exact extent and underlying pathophysiology of this phenomenon remain unclear.

The aim of this study was to evaluate contrast-enhanced DECT chest examinations regarding pulmonary perfusion patterns and pulmonary opacities in patients with RT-PCR-confirmed COVID-19 disease.

## 2. Materials and Methods

### 2.1. Study Design

This monocentric, retrospective study was approved by the institutional review board with a waiver of informed consent (project number 609/2020BO; 31 July 2020). All the study procedures were conducted in accordance with the ethical standards, as laid down in the 1964 Declaration of Helsinki and its later amendments. The inclusion criteria were a positive RT-PCR test for COVID-19 and a contrast-enhanced DECT examination of the chest, with a maximum of 25 days after a positive RT-PCR test. Adult patients were identified via the institutional radiology information system. Fourteen confirmed COVID-19 patients with 24 DECT examinations performed between April and May 2020 were found and displayed the final study group.

### 2.2. DECT Imaging Acquisition Parameters

All the DECT examinations were acquired using a dual-source scanner (Siemens SOMATOM Force, Siemens Healthineers, Erlangen, Germany). After scout acquisition, imaging was conducted in a supine position and cranio-caudal scanning direction. Image datasets were acquired in portal-venous phase with a delay of 80 s after the administration of a non-ionic iodine contrast agent (iomeprol, Imeron 400, Bracco, Milan, Italy), which was based on the patients’ body weight (1 mL/kg), and followed by a saline flush using a flow rate of 2.0 mL/s. The dual-energy imaging protocol consisted of a collimation of 0.6 mm, as well as of a tube voltage of 100/Sn150 kV and a reference tube current of 190/95 mAs using automatic tube current modulation (CARE Dose, Siemens Healthineers, Erlangen, Germany). The mean CTDIvol was 15.7 ± 7.0 mGy. Imaging reconstruction was performed using a soft tissue kernel (Qr40) in axial and coronal plane with a slice thickness of 1.5 mm.

### 2.3. DECT Imaging Analysis

Image evaluation was performed independently by two radiologists with four and seven years of experience, respectively. Images were assessed on a dedicated workstation (syngo.via, Siemens Healthineers, Erlangen, Germany). Both radiologists were blinded to clinical data. The image datasets were analyzed regarding image quality using a Likert scale ranging from 1 to 4 for the overall image quality (1 = non-diagnostic; 2 = diagnostic, but severely impaired image quality; 3 = diagnostic, slightly impaired image quality; 4 = diagnostic, excellent image quality), for the presence and extent of artifacts (1: non diagnostic, with major artifacts; 2 = diagnostic, moderate artifacts; 3 = diagnostic, minor artifacts; 4 = diagnostic, no artifacts), and for the sharpness of pulmonary vessels (1 = excessive motion and pulsation artifacts with blurred contours of pulmonary vessels; 2 = moderate motion and pulsation artifacts with blurred contours of pulmonary vessels; 3 = slight motion and pulsation artifacts with recognizable contours of pulmonary vessels; 4 = no motion and pulsation artifacts with sharp contours of pulmonary vessels). Furthermore, the extent of perfusion defects within the pulmonary lobes were characterized using a Likert scale ranging from 1 to 4 (1 ≤ 25% perfusion defect; 2 = 26–50% perfusion defect; 3 = 51–75% perfusion defect; 4 ≥75% perfusion defect). Table 1 shows the scoring system for opacities and perfusion defects.

Finally, the presence of mosaic perfusion patterns was analyzed in lung areas without consolidation and GGO on a nominal scale.

### 2.4. Automatic Analysis of Lung Opacities

To compare the extent of perfusion defects with lung opacities, all the DECT datasets were processed using a software prototype (CT Pneumonia Analysis 2.1.2, Siemens Healthineers, Erlangen, Germany), automatically providing the percentage of lung opacities (consolidation and GGO) within each lobe using three-dimensional segmentations. The extent of opacities was classified using the same Likert scale as described for perfusion defects above (Table 1). Additionally, the lung volume in ml was given for each lobe.

### 2.5. Statistical Analysis

Proprietary statistical software was used for evaluation (SPSS Statistics Version 26, IBM, Armonk, NY, USA). Continuous variables are displayed with the mean ± standard deviation (SD). Non-parametric data are displayed using the median and interquartile range (IQR) in parentheses. For paired data, the dependent *t*-test and the paired Wilcoxon signed rank-test were applied. *p*-values below 0.05 were regarded as significant.

## 3. Results

### 3.1. Characteristics of the Study Group

All 24 examinations could be evaluated successfully. The mean patient age at examination date was 57 ± 14 years (range, 32–80 years). The median time between RT-PCR and CT examination was 8 days (3–16 days). Indications for CT imaging were most often clinical deterioration and search for an inflammatory origin (*n* = 15) and the suspicion of bleeding (*n* = 4). Further details are displayed in Table 2.

**Figure 1:**
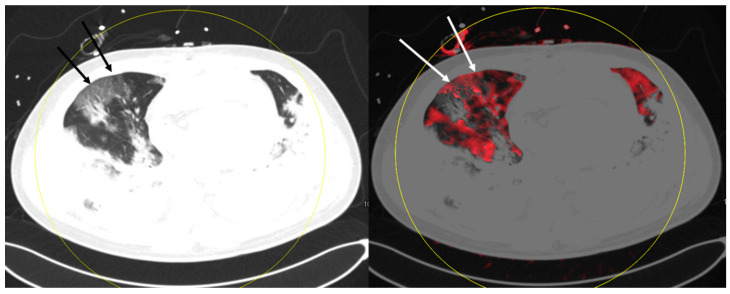
This patient shows mosaic perfusion of ground glass opacities in the right lung with perfusion defects (white arrows) in some areas of ground glass opacities (black arrows). Furthermore, mosaic perfusion of areas without ground glass opacities is present.

**Figure 2:**
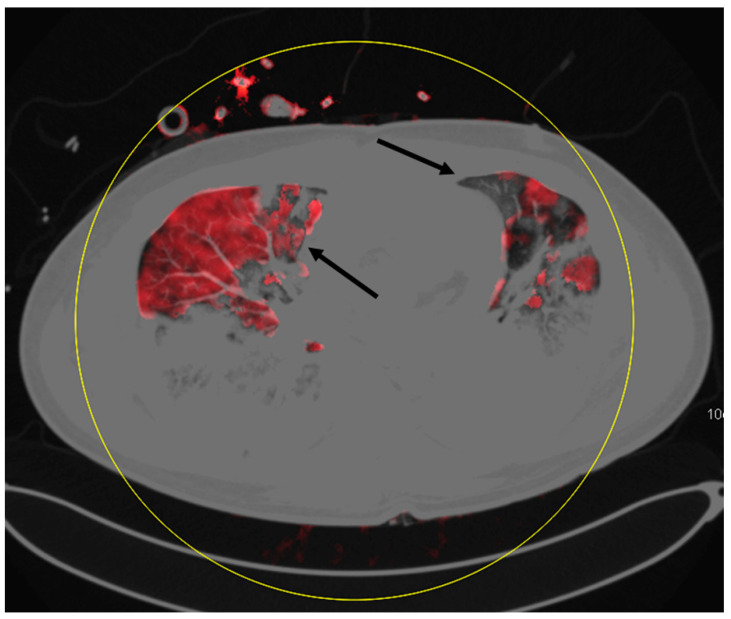
This example displays areas of ground glass opacities with perfusion defects (black arrow left lung) and ground glass opacities with normal perfusion (black arrow right lung).

**Figure 3:**
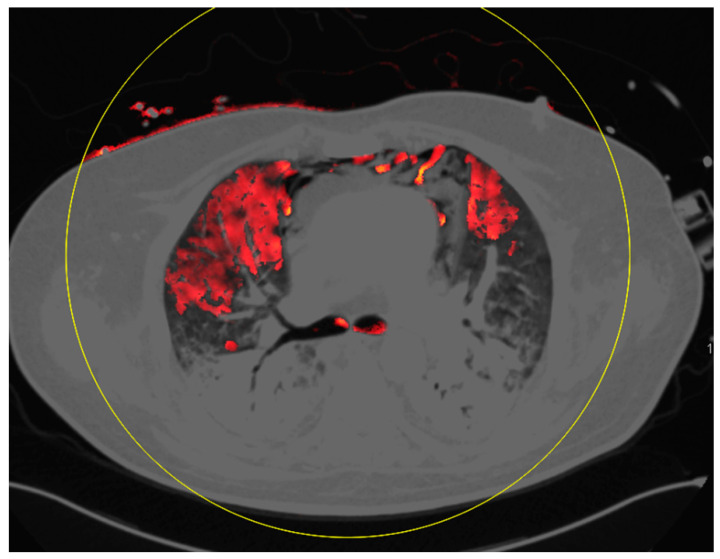
Large areas of perfusion defects in both lungs with ground glass opacities.

The main pulmonary findings were GGO in all 24 examinations, consolidation in 22 examinations, pleural effusion in 17 examinations, fibrotic streaks in three examinations, bronchiectasis in two examinations, and pneumothorax in one examination. Furthermore, mediastinal lymphadenopathy was found in one examination. Non-pulmonary-related secondary findings were the thrombosis of jugular veins in five examinations, intercostal active arterial bleeding in one examination, and aneurysm of the ascending aorta in one examination. None of the examinations revealed macroscopic signs of pulmonary embolism. Mosaic perfusion patterns were found in 19 examinations. These perfusion defects were located in subpleural as well as in more central lung parenchyma parts. Figure 1, Figure 2 and Figure 3 show examples of mosaic perfusion and perfusion defects.

The mean lung volume according to automatic evaluation was 2988 ± 1178 mL. Further details on the study group are displayed in Table 3. Lung volume was negatively correlated with perfusion defects, with a Pearson correlation coefficient of −0.67 for the left lung and −0.60 for the right lung.

### 3.2. Image Quality Assessment

Overall, the inter-reader agreement between both readers was excellent (Cohen’s kappa of 0.97). The image quality regarding artifacts was rated by both readers with a median of 4 (IQR 4–4). The sharpness of lung vessels was rated by both readers with a median of 4 (IQR 3–4). The overall image quality was evaluated to be excellent with a median of 4 (IQR 4–4) by both readers (Table 4).

### 3.3. Perfusion Defects and Automatic Opacity Score Analysis

**Figure 4:**
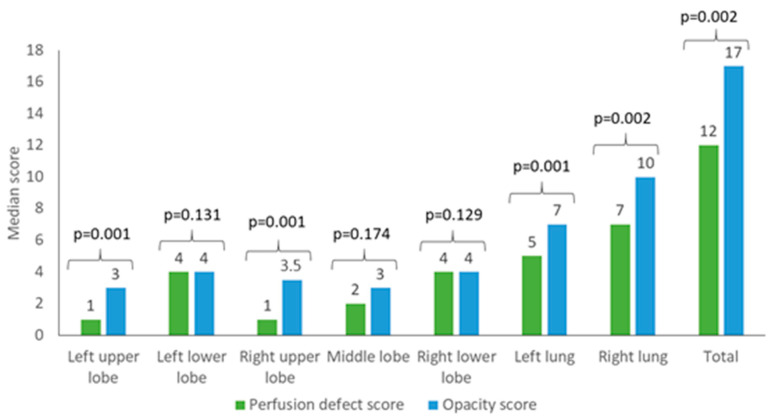
Median perfusion defect scores of reader 1 are displayed in green. Automatic opacity score is shown in blue.

**Figure 5:**
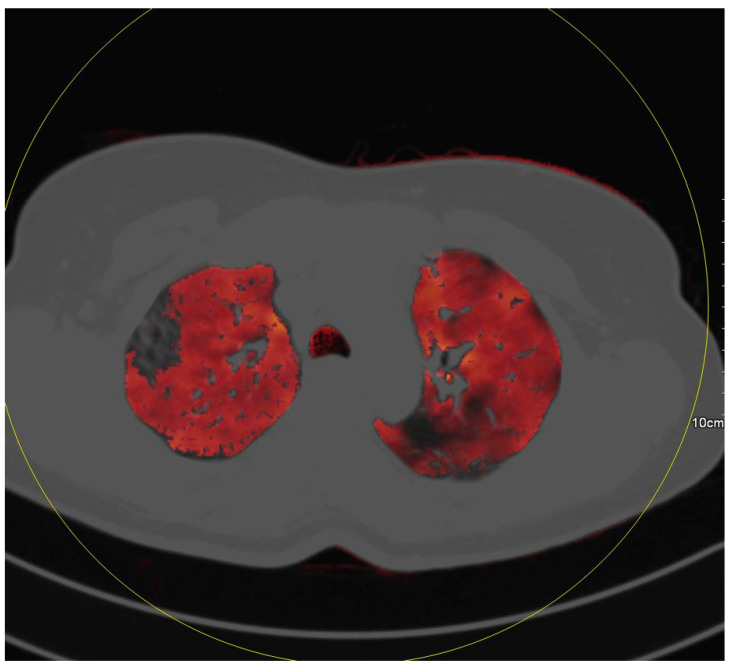
This patient shows only slight perfusion defects as well as opacities in both upper lobes (perfusion defect score 1; opacity score 1).

**Figure 6:**
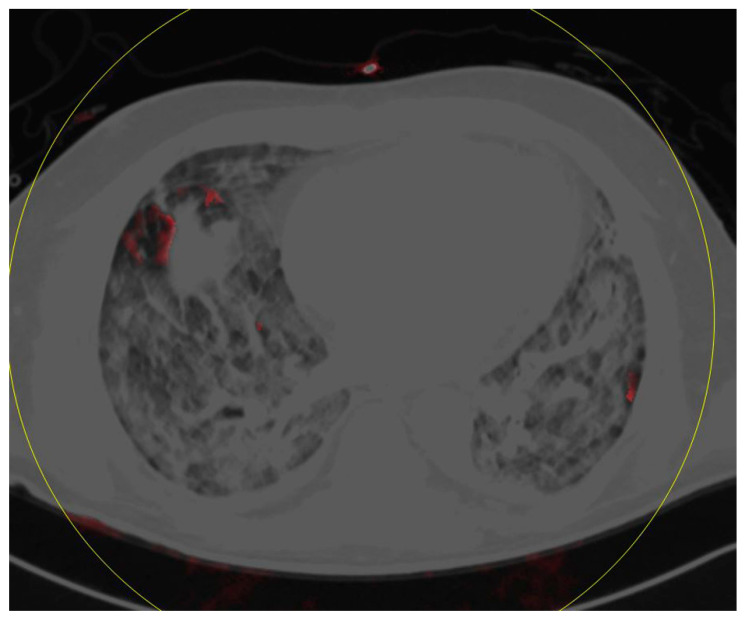
This patient shows extensive perfusion defects as well as opacities in both lower lobes (perfusion defect score 4; opacity score 4).

Due to the excellent inter-reader agreement, only the results of reader 1 are displayed in the following. The results of reader 2 are shown in Table 5. The left upper lobe showed significantly more visible opacities than perfusion defects in DECT imaging with a median of 3 (IQR 2–4) compared to 1 (IQR 1–4) (*p* = 0.001). Similar results were obtained for the right upper lobe with a median opacity score of 3.5 (IQR 3–4) compared to a median perfusion defect score of 1 (IQR 1–3) (*p* = 0.001). However, there was no significant difference regarding the lower lobes and the middle lobe. The left lower lobe was rated with a median opacity score of 4 (IQR 4–4) versus a median perfusion defect score of 4 (IQR 3–4) (*p* = 0.131). The middle lobe was rated with a median opacity score of 3 (IQR 1–4) compared to a median perfusion defect score of 2 (IQR 1–3) (*p* = 0.174). The right lower lobe was evaluated with a median opacity score of 4 (IQR 4–4) and a median perfusion defect score of 4 (3–4) (*p* = 0.129). The addition of the single lobe scores resulted in a median score of 5 (IQR 4–8) for perfusion defects of the left lung versus a median opacity score of 7 (IQR 6–8) (*p* = 0.001). The right lung resulted in a median perfusion defect score of 7 (IQR 5–11) compared to a median opacity score of 10 (IQR 8–11) (*p* = 0.002). In total, for both lungs the median perfusion defect score was 12 (IQR 9–18), versus the median opacity score of 17 (IQR 15–19) (*p* = 0.002). Figure 4 shows an analysis of the perfusion defects and opacities of reader 1. Figure 5 and Figure 6 display examples for different scores.

## 4. Discussion

This study investigated the relationship between pulmonary perfusion defects and pulmonary opacities in patients with confirmed COVID-19 disease and without signs of pulmonary embolism. The evaluation of the imaging datasets showed a significantly higher proportion of opacities overall as well as in the left and right upper lobe compared to perfusion defects. However, there was no significant difference between the opacities and perfusion defects in the middle lobe and in the left and right lower lobe. Furthermore, in most examinations mosaic perfusion patterns were found.

The morphological findings of consolidations and GGO in COVID-19 patients are in line with those of previous publications [7,10,11,13]. However, there are only sparse data available regarding pulmonary perfusion defects in COVID-19 patients. One of the main findings of our study is that there is a higher percentage of opacities compared to perfusion defects, indicating the maintained perfusion of areas with GGO. Furthermore, significant differences between the extent of perfusion defects and opacities were found in the upper lobes. This might be explained by the craniocaudal shift of perfusion, with increased blood flow in the basal lung areas. Another important finding of our study consists of perfusion defects in pulmonary areas without GGO. Mosaic perfusion patterns can be caused by two different mechanisms, according to the Euler–Liljestrand mechanism in pulmonary hypoxia: The first mechanism consists of air trapping and a consecutive limited participation of these lung areas in blood oxygenation. The second mechanism is based on the pathologies of supplying blood vessels, resulting in limited blood supply. In previous studies, an increased incidence of pulmonary embolism was reported in a significant proportion of COVID-19 patients [19,31]. However, in our study no patient revealed macroscopic pulmonary embolism, indicating other responsible causes of the perfusion defects detected by DECT. As no hyperlucent areas could be matched with perfusion defects, the most logical explanation for perfusion defects is related to issues of microperfusion pathologies. Similar results for mosaic perfusion and regional perfusion changes have already been reported by Lang et al. in COVID-19 patients, as well as generally in acute respiratory distress syndrome [11,30,32]. Thromboembolic pulmonary and non-pulmonary incidents have also previously been described by Oudkerk et al. [20]. However, as no macroscopic thromboembolic incidents were evident in our study cohort, microembolic issues seem to play a vital role. Microperfusion-related injuries have also previously been demonstrated in autopsy studies, with findings of endothelial injuries of vessels, alveolar capillary microthrombosis, and neoangiogenesis [17,18]. Other studies have suggested the presence of fibrin deposition within alveolar capillaries and the presence of megakaryocytes as a possible source of perfusion disorders [33]. Of course, as in sepsis due to other pathogens, cytokine storms and hypercytokinemia could also display significant parameters and be one piece of the puzzle [33,34].

As already mentioned, some GGO have revealed perfusion defects, whereas other GGO have revealed normal perfusion compared to the neighboring lung tissue. However, the pathophysiological background of this finding remains unclear. The preserved perfusion of GGO might display a positive prognostic parameter, whereas the reduced perfusion of GGO might indicate severe or irreversible lung parenchyma damage. Long-term follow up scans will be necessary in order to investigate the consequences of these findings. Further studies will also be necessary to compare the effects of lung perfusion in GGO due to other causes—e.g., infectious diseases of other pathogens or trauma. So far, the data regarding these areas are still sparse.

Our study has several limitations. The number of patients as well as the number of examinations is very small. Therefore, this investigation displays preliminary results only. However, due to the first appearance ever of COVID-19, the investigation of a larger patient cohort was not possible. Furthermore, all our DECT examinations were performed in the portal-venous phase. This might hamper the identification of macroscopic pulmonary embolism. However, we think that the acquisition of a portal-venous phase displays also a unique strength of our study, showing a prolonged perfusion deficit in some lung areas that cannot be compensated by collaterals. Ideally, further studies should be performed with multi-phase DECT acquisitions to investigate perfusion defects independently of the contrast phase.

## 5. Conclusions

In conclusion, in our study we found larger proportions of lung opacities compared to perfusion defects. Furthermore, most studies have revealed patterns of mosaic perfusion in areas with and without GGO. Further studies will be necessary to investigate the pathophysiological background of GGO with maintained perfusion compared to GGO with reduced perfusion, especially regarding long-term lung damage and prognosis.

## Figures and Tables

**Table 1:** Evaluation scores.

Characteristics	Values
Opacity score	1: ≤25% lung opacity
	2: 26–50% lung opacity
	3: 51–75% lung opacity
	4: ≥75% lung opacity
Perfusion defect score	1: ≤25% lung perfusion defect
	2: 26–50% lung perfusion defect
	3: 51–75% lung perfusion defect
	4: >75% lung perfusion defect

**Table 2:** Characteristics of the study group.

Characteristics	Values
Examinations	*n* = 24
Patients	*n* =14 (male: *n* = 11)
Invasive ventilation	*n* = 20
Mean age ± std	55 ± 16 years
Range	32–80 years
Median time RT-PCR—DECT	8 days (3–16 days)
Median time between first CT and follow-up	13 days (9–18 days)
*Indications for imaging*	
Clinical deterioration and search for inflammatory origin	*n* = 15
Suspicion of bleeding	*n* = 4
Follow-up of pulmonary status	*n* = 2
Suspicion of cervical thrombosis	*n* = 1
Trauma	*n* = 1
Staging	*n* = 1

**Table 3:** Imaging findings and lung volume analysis.

Characteristics	Values
*Main pulmonary findings*	
Ground glass opacity	*n* = 24
Consolidation	*n* = 22
Mosaic perfusion pattern	*n* = 19
Pleural effusion	*n* = 17
Fibrotic streaks	*n* = 3
Bronchiectasis	*n* = 2
Pneumothorax	*n* = 1
Mediastinal lymphadenopathy	*n* = 1
*Secondary non-pulmonary findings*	
Thrombosis of jugular veins	*n* = 5
Thoracic bleeding	*n* = 1
Aortic aneurysm	*n* = 1
*Lung volume*	
Left upper lobe	829 ± 384 mL
Left lower lobe	473 ± 310 mL
Right upper lobe	662 ± 304 mL
Middle lobe	324 ± 178 mL
Right lower lobe	700 ± 253 mL
Left lung	1302 ± 588 mL
Right lung	1685 ± 615 mL
Total	2988 ± 1178 ml

**Table 4:** Image quality assessment (median (interquartile range; IQR)).

Characteristics	Reader 1	Reader 2
Artifacts	4 (IQR 4–4)	4 (IQR 4–4)
Sharpness of lung veseels	4 (IQR 3–4)	4 (IQR 3–4)
Overall image qualtiy	4 (IQR 4–4)	4 (IQR 4–4)

**Table 5:** Median (interquartile range; IQR) perfusion defect scores of readers 1 and 2 and the automatic opacity score.

	Perfusion Defects Reader 1	Automatic Opacity Score	*p*-Value
	Reader 2		
Left upper lobe	1 (IQR 1–4)	3 (IQR 2–4)	**0.001**
	1 (IQR 1–4)		**0.001**
Left lower lobe	4 (IQR 3–4)	4 (IQR 4–4)	0.131
	4 (IQR 3–4)		0.197
Right upper lobe	1 (IQR 1–3)	3.5 (IQR 3–4)	**0.001**
	1 (IQR 1–3)		**0.001**
Middle lobe	2 (IQR 1–3)	3 (IQR 1–4)	0.174
	2 (IQR 1–3)		0.174
Right lower lobe	4 (IQR 3–4)	4 (IQR 4–4)	0.129
	4 (IQR 2.5–4)		0.131
Left lung	5 (IQR 4–8)	7 (IQR 6–8)	**0.001**
	5 (IQR 4–8)		**0.001**
Right lung	7 (IQR 5–11)	10 (IQR 8–11)	**0.002**
	7 (IQR 5–11)		**0.001**
Total	12 (IQR 9–18)	17 (IQR 15–19)	**0.002**
	12.5 (IQR 9–18)		**0.002**

Bold: the significant *p*-values.
